# TRIM25‐Mediated Ubiquitination and Degradation of SOX8 Promotes Ligament Fibroblast Osteogenic Differentiation and Regulates OPLL Progression by Inhibiting OSR2 Transcription

**DOI:** 10.1002/jsp2.70112

**Published:** 2025-09-05

**Authors:** Zhenqiang Wang, Yifan Tang, Changjiang Gu, Minming Lu, Ziheng Wei, Quanwei Zhou, Shengyuan Zhou, Xiongsheng Chen

**Affiliations:** ^1^ Spine Center, Department of Orthopaedics Changzheng Hospital, Naval Medical University (Second Military Medical University) Shanghai People's Republic of China; ^2^ Department of Orthopaedics Quanzhou First Hospital Affiliated to Fujian Medical University Quanzhou Fujian People's Republic of China; ^3^ Department of Orthopaedics Shanghai General Hospital, Shanghai Jiao Tong University School of Medicine Shanghai People's Republic of China; ^4^ Department of Pain Medicine Wenjiang Hospital of Sichuan Provincial People's Hospital Chengdu People's Republic of China; ^5^ Department of Spinal Surgery Yangzhi Rehabilitation Hospital (Shanghai Sunshine Rehabilitation Center), Tongji University School of Medicine Shanghai People's Republic of China

**Keywords:** OPLL, OSR2, SOX8, TRIM25, ubiquitination

## Abstract

**Background:**

Ossification of the posterior longitudinal ligament (OPLL) is a pathological condition characterized by ectopic ossification of spinal ligaments, primarily driven by abnormal osteogenic differentiation of ligament fibroblasts with stem cell‐like properties. The SOX transcription factor family is crucial in regulating cell stemness and differentiation. Among them, SOX8 is known to influence osteoblast differentiation, but its role in OPLL remains unclear.

**Methods:**

SOX8 expression was analyzed in non‐OPLL and OPLL ligament tissues and cells. Its role in osteogenic differentiation was assessed using ALP/Alizarin Red staining, qPCR, Western blotting, and subcutaneous ectopic ossification models in nude mice. Mass spectrometry and co‐immunoprecipitation identified SOX8‐interacting E3 ubiquitin ligases, with ubiquitination assays assessing their effects on SOX8 stability. RNA‐seq, GTRD analysis, and dual‐luciferase reporter assays revealed SOX8 target genes. Functional recovery experiments were conducted to explore the role of these interactions in the osteogenic differentiation of ligament fibroblasts.

**Results:**

SOX8 expression was downregulated in OPLL ligament tissues and cells. Functional analyses showed that SOX8 inhibits osteogenic differentiation of ligament fibroblasts both in vitro and in vivo. Mechanistically, TRIM25, an E3 ubiquitin ligase, was found to interact with SOX8, promoting its ubiquitination and degradation. Rescue experiments showed that SOX8 knockdown or overexpression reversed the osteogenic effects of TRIM25 knockdown or overexpression in ligament fibroblasts. Additionally, OSR2 was identified as a transcriptional target of SOX8, with SOX8 promoting OSR2 transcription. OSR2 knockdown negated the inhibitory effects of SOX8 overexpression on osteogenic differentiation.

**Conclusions:**

SOX8 serves as a critical negative regulator of osteogenic differentiation in ligament fibroblasts. TRIM25 promotes ectopic ossification in OPLL by enhancing SOX8 ubiquitination and degradation, while SOX8 inhibits osteogenic differentiation through transcriptional activation of OSR2. These findings highlight the TRIM25/SOX8/OSR2 axis as a key regulator in OPLL ectopic ossification, suggesting it to be a potential target for non‐surgical treatment.

## Graphical abstract

SOX8 is downregulated in OPLL and inhibits the osteogenic differentiation of ligament fibroblasts. TRIM25 promotes the ubiquitination and degradation of SOX8, while SOX8 transcriptionally activates OSR2 to suppress osteogenesis. The TRIM25/SOX8/OSR2 axis represents a key regulatory mechanism and a potential therapeutic target for OPLL.

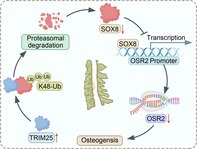

## 1 Introduction

Ossification of the posterior longitudinal ligament (OPLL) is a pathological ectopic ossification of the posterior longitudinal ligament in the spine, with approximately 70% of cases occurring in the cervical spine [1]. This condition exhibits a higher prevalence in Asian populations, elderly individuals, and males [2]. As ectopic ossification progresses, patients develop symptoms of spinal cord and nerve compression, significantly impairing their ability to work and quality of life. Currently, surgical intervention remains the primary treatment for OPLL, as no effective pharmacological therapies or preventive measures to halt disease progression have been established. However, OPLL surgery is technically demanding and associated with high risks, including complications such as spinal cord injury, cerebrospinal fluid leakage, and postoperative recurrence of ossification [3, 4, 5]. Therefore, elucidating the molecular mechanisms underlying OPLL pathogenesis is critical for developing targeted non‐surgical therapies.

OPLL is recognized as a multifactorial disease influenced by both genetic and environmental factors [6, 7, 8]. Notably, ligament fibroblasts derived from the posterior longitudinal ligament exhibit stem‐like characteristics and possess multidirectional differentiation potential, making their osteogenic differentiation a central focus in OPLL research [9, 10, 11]. The SOX protein family consists of transcription factors with a highly conserved high‐mobility group (HMG) domain, which are involved in regulating numerous biological processes like stem cell maintenance and neurogenesis, and are essential for the development and upkeep of the skeletal system [12, 13, 14, 15, 16]. For instance, SOX11 can enhance the self‐renewal and osteogenic differentiation of mesenchymal stem cells under the induction of Wnt7b [17], whereas SOX9 supports chondrogenesis but serves as a negative regulator of endochondral ossification, thus hindering bone formation [18, 19]. Additionally, SOX8, a member of the SOX E subgroup, has been reported to synergize with SOX9 and SOX10 in response to BMP signaling to drive chondrogenesis in limb mesoderm [20]. Intriguingly, SOX8 has been recognized as a negative regulator of osteoblast differentiation [21], suggesting its potential involvement in OPLL‐associated ectopic ossification. However, whether SOX8 plays a role in OPLL ectopic ossification and its underlying molecular mechanisms remain unexplored.

Ubiquitination, a critical post‐translational modification, has emerged as a key regulatory mechanism in bone formation and ectopic ossification [22, 23, 24]. This pathway, governed by ubiquitin‐activating enzymes (E1), ubiquitin‐conjugating enzymes (E2), and ubiquitin ligases (E3), modulates the stability, activity, and localization of proteins [25, 26]. Recent studies have highlighted the importance of ubiquitination in OPLL development, with specific proteins such as CXCL7 and Cx43 being regulated through ubiquitin‐dependent mechanisms [27, 28]. Intriguingly, several SOX family members, including SOX2 and SOX9, are known to be regulated by ubiquitination [29, 30], suggesting a potential link between SOX8 and ubiquitin‐mediated pathways in OPLL. However, whether SOX8 is subject to ubiquitination and how this process influences OPLL pathogenesis remain to be elucidated.

In this research, we intended to investigate the function of SOX8 in the pathogenesis of OPLL and its unique regulatory mechanism. The expression of SOX8 in posterior longitudinal ligament tissues and fibroblasts from non‐OPLL and OPLL patients was examined. We also studied the role of SOX8 in the osteogenic differentiation of ligament fibroblasts and subcutaneous ectopic ossification. Additionally, we investigated the effect of ubiquitination on SOX8 protein levels and its impact on OPLL ectopic ossification. We found that the E3 ubiquitin ligase TRIM25 interacts with SOX8 and promotes its ubiquitination‐mediated proteasomal degradation. Through the use of transcriptome sequencing, GTRD analysis, and dual‐luciferase reporter assays, we identified OSR2 as a downstream target of SOX8, which enhances OSR2 promoter activity to modulate gene transcription, thereby influencing OPLL ectopic ossification. Collectively, our findings highlight the critical role of the TRIM25/SOX8/OSR2 axis in the pathogenesis of OPLL, offering new therapeutic targets for this challenging condition.

## 2 Materials and Methods

### 2.1 Collection of Cervical Posterior Longitudinal Ligament (PLL) Clinical Samples and Isolation and Culture of Ligament Fibroblasts

In this study, 12 OPLL patients and 12 non‐OPLL patients (with cervical spine injury) were included. Specimens were obtained during anterior cervical ossified posterior longitudinal ligament en bloc resection (ACOE) or anterior cervical subtotal corpectomy surgeries performed on OPLL or cervical spine injury patients. Patients with osteoporosis, tumors, infections, or a history of drug abuse were excluded. All patients provided informed consent before undergoing surgery. During the surgical procedure, cervical posterior longitudinal ligament specimens were collected, placed in DMEM medium supplemented with serum, and kept on ice. The specimens were transported to the laboratory under low‐temperature conditions. In a sterile environment, the ossified tissue surrounding the ligament was trimmed into approximately 1 to 2 mm^3^ pieces and cultured in a temperature‐controlled incubator for cell adherence. Once the cells migrated from the tissue and reached 70% confluence, the tissue was discarded, and the cells were passaged. Subsequent experiments employed ligament fibroblasts from passages P2 through P4.

### 2.2 Cultivation of 293T Cells

Sourced from the Cell Bank at the Chinese Academy of Sciences in Shanghai, China, the HEK 293T cells were cultured in Gibco DMEM medium with 10% FBS and 1% penicillin/streptomycin in a temperature‐controlled incubator.

### 2.3 Lentiviral and Plasmid Transfection

A multiplicity of infection (MOI) of 50 was used to infect OPLL ligament fibroblasts with lentivirus (SOX8, TRIM25, OSR2). Stable transfected cells were selected using puromycin and either cryopreserved or passaged for further use. At 80%–90% confluence, cells underwent transfection with Lipofectamine 8000 reagent, Opti‐MEM medium, and plasmids. Cells were gathered 48 h post‐transfection for additional experiments.

### 2.4 Osteogenic Differentiation Induction

The addition of osteogenic differentiation medium to cells at 80%–90% confluence initiated osteogenesis. Osteogenic differentiation was monitored regularly under a microscope. The medium used for osteogenic induction included DMEM, 10% FBS, 1% penicillin/streptomycin, ascorbic acid, β‐glycerophosphate, and dexamethasone.

### 2.5 Western Blot

The cells were washed with PBS after the medium was removed once they grew to the right density. Cells underwent lysis on ice for 20 min using a buffer with protease and phosphatase inhibitors. The lysates were centrifuged at 4°C at a speed of 12,000 rpm for 5 min, and the supernatant was collected for protein quantification with a BCA protein assay kit from Beyotime. The proteins were combined with SDS sample buffer and heated at 95°C for 10 min prior to their separation by SDS‐PAGE and subsequent transfer to a PVDF membrane. A quick protein blocking solution was used to treat the membrane, which was then incubated with primary and secondary antibodies. The ECL detection system (EpiZyme, SQ202) was employed to visualize the protein bands. The experiments involved primary antibodies listed in Table S1, with GAPDH acting as an internal control.

### 2.6 Real‐Time qPCR

After extracting total RNA from cells, its concentration and purity were checked with a Nanodrop 2000. Reverse transcription was used to synthesize cDNA, and PCR amplification was done with SYBR GREEN. GAPDH served as an internal reference. Table S2 contains the relevant primer sequences.

### 2.7 Alkaline Phosphatase (ALP) Staining and Alizarin Red Staining

To assess osteogenic differentiation, ALP and Alizarin Red staining were applied. Cells were washed with PBS, fixed in 4% paraformaldehyde, and stained using an ALP kit (Beyotime, China) and Alizarin Red S (Cyagen, China). Stained cells were observed and photographed.

### 2.8 Immunoprecipitation and Proteomics (LC–MS/MS)

Protein lysates were first exposed to SOX8, TRIM25, or IgG antibodies at room temperature for an hour, then incubated with Protein A + G magnetic beads (Beyotime, China) overnight at 4°C. The protein‐bead complexes were then boiled at 95°C to elute proteins, which were subsequently analyzed by Western blot. In accordance with the manufacturer's instructions, cell protein extracts were incubated overnight with Flag/His magnetic beads (Beyotime, China) at 4°C for immunoprecipitation. The proteins that were bound were eluted at 95°C and analyzed using Western blot. Additionally, the antigen–antibody‐bead complexes were used for proteomic analysis to evaluate the isolated immunoprecipitates.

### 2.9 Immunofluorescence Staining

Ligament fibroblasts were cultured on sterile glass coverslips. After reaching the target confluence, the cells were fixed with 4% paraformaldehyde, permeabilized, blocked, and incubated with antibodies. DAPI was utilized for staining the nuclei, and the slides were prepared for inspection using a fluorescence microscope.

### 2.10 Ubiquitination Detection

Immunoprecipitation with an anti‐SOX8 antibody was conducted on ligament fibroblast lysates to examine endogenous SOX8 ubiquitination, followed by Western blot using an anti‐ubiquitin antibody. HEK 293T cells were co‐transfected with Flag‐SOX8, His‐TRIM25, and HA‐Ub for exogenous ubiquitination, with detection performed via immunoprecipitation and Western blot.

### 2.11 Dual‐Luciferase Reporter Assay

Lipofectamine 8000 was used to co‐transfect 293T cells with the OSR2 promoter luciferase reporter plasmid, a SOX8 overexpression plasmid, and the corresponding control plasmids. Luciferase activity was evaluated 48 h later using the Dual‐Luciferase Reporter Assay System (Promega, E1910), with firefly luciferase activity being standardized against Renilla luciferase activity. The data were normalized accordingly.

### 2.12 In Vivo Bone Formation Assay

Approval for all animal experiments was granted by the Committee on Ethics of Medicine, Navy Medical University, PLA. Five‐week‐old BALB/c nude mice participated in the study. Fibroblasts from ligaments, modified with either lentivirus or a control virus, were grown together with Bio‐Oss collagen scaffolds (Geistlich, Switzerland) and placed under the skin in the right groin area of nude mice (three mice per group).

### 2.13 Micro‐CT Scanning

Images from micro‐CT scanning, performed with a Skyscan 1176 (Bruker, USA), were processed using NRecon software for the purposes of 3D reconstruction and quantification.

### 2.14 Immunohistochemistry Staining

After decalcification and dehydration, tissues were embedded in paraffin and stained with hematoxylin and eosin (H&E) for general histological analysis. Standard protocols were followed for immunohistochemistry using primary antibodies (SOX8, TRIM25, OSR2, COL1A1, RUNX2) and secondary antibodies, and the sections were examined with an optical microscope. Details regarding the primary antibodies used in the experiments are available in Table S1.

### 2.15 Statistical Analysis

Results are shown as mean ± standard deviation (SD), and statistical analyses were carried out with SPSS 22.0 software on Windows. To compare two groups, a *t*‐test was applied. For multiple group comparisons, one‐way ANOVA was used. A *p*‐value below 0.05 indicated statistical significance.

## 3 Results

### 3.1 Decreased Expression of SOX8 in Posterior Longitudinal Ligament and Fibroblasts of OPLL Patients

**FIGURE 1:**
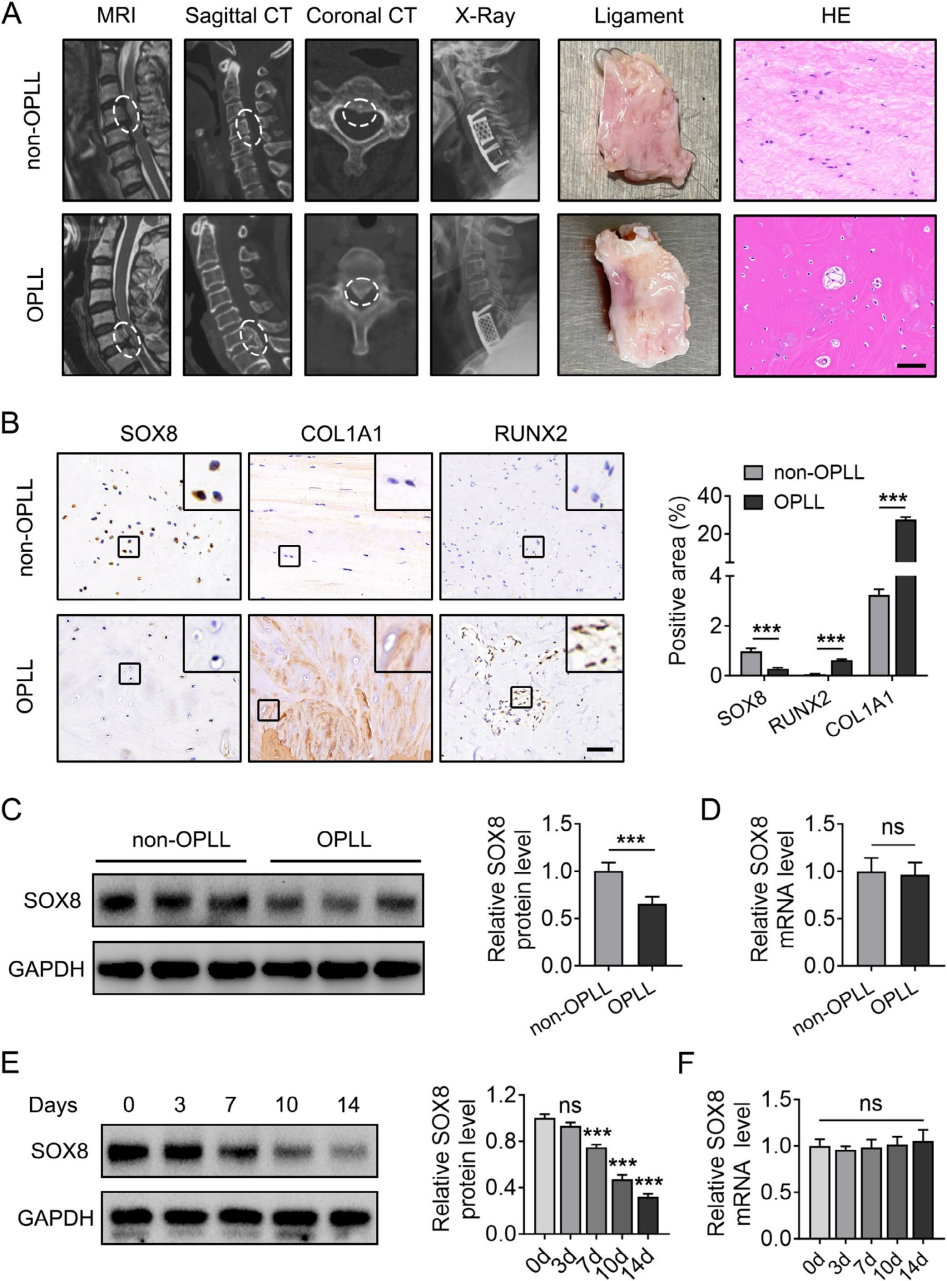
Decreased SOX8 expression in PLL tissues and fibroblasts from OPLL patients. (A) Representative preoperative and postoperative imaging, along with PLL specimens, and H&E staining of PLL tissues from non‐OPLL and OPLL patients (scale bar, 100 μm). (B) Immunohistochemical staining of SOX8, COL1A1, and RUNX2 in PLL tissues from non‐OPLL and OPLL patients (scale bar, 100 μm). (C, D) Western blot and qPCR analyses of SOX8 expression in ligament fibroblasts from non‐OPLL and OPLL patients (*n* = 6). (E, F) Western blot and qPCR analyses of SOX8 expression during osteogenic differentiation of ligament fibroblasts. GAPDH was used as the internal control. Data are presented as mean ± standard deviation. *p < 0.05, **p < 0.01, ***p < 0.001 (all quantitative analyses were performed in triplicate unless otherwise specified).

To investigate the role of SOX8 in OPLL‐associated ectopic ossification, cervical posterior longitudinal ligament (PLL) specimens were collected from 12 OPLL patients and 12 non‐OPLL patients (with cervical spine trauma). Table S3 summarizes the baseline characteristics of the two groups, and Figure 1A shows typical imaging results from before and after the operation. Complete PLL specimens were obtained through anterior cervical ossified posterior longitudinal ligament en bloc resection (ACOE) or anterior cervical subtotal corpectomy [31] (Figure 1A). We first examined SOX8 expression levels in PLL tissues and fibroblasts from OPLL and non‐OPLL patients. Immunohistochemical staining revealed significantly reduced SOX8 expression in PLL tissues from OPLL patients compared to those from non‐OPLL patients (*p* < 0.05) (Figure 1B). Consistent with previous studies [10, 32, 33], the expression levels of osteogenesis‐related genes (COL1A1 and RUNX2) were markedly elevated in OPLL PLL tissues compared to non‐OPLL tissues (Figure 1B). Fibroblasts were extracted and grown from PLL tissues of both OPLL and non‐OPLL patients using the tissue block adhesion culture technique (Figure S1A–C). Western blot analysis confirmed significantly lower SOX8 protein levels in fibroblasts from OPLL patients (Figure 1C). Interestingly, qPCR showed no significant difference in SOX8 mRNA levels between the two groups (Figure 1D). Further investigation revealed a progressive decline in SOX8 protein levels during the osteogenic differentiation of ligament fibroblasts (Figure 1E), whereas SOX8 mRNA levels remained unchanged during differentiation (Figure 1F). These findings suggest that the reduction of SOX8 in OPLL may be regulated at the post‐translational level. Taken together, these results demonstrate a decreased expression of SOX8 in PLL tissues and fibroblasts of OPLL patients, implicating its potential role in the process of ectopic ossification in OPLL.

### 3.2 SOX8 Negatively Regulates Osteogenic Differentiation of Ligament Fibroblasts In Vitro

**FIGURE 2:**
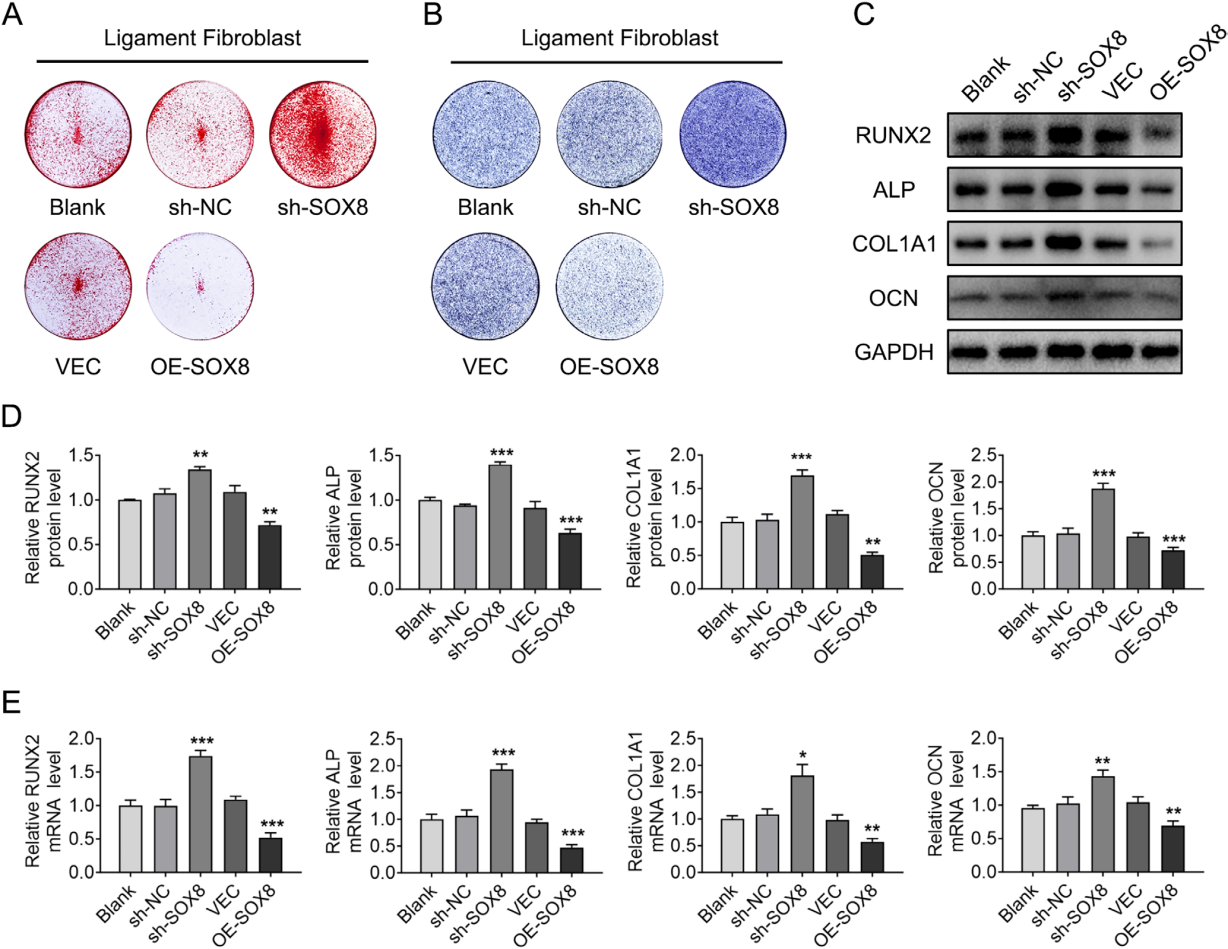
SOX8 inhibits the osteogenic differentiation of ligament fibroblasts in vitro. (A, B) Alizarin Red staining (A) and ALP staining (B) show the inhibitory effect of SOX8 on the osteogenic differentiation of ligament fibroblasts. (C, D) Western blot analysis (C) and corresponding densitometric quantification (D) of osteogenic marker gene expression are presented. (E) qPCR analysis of osteogenic marker genes. GAPDH was used as the internal control. For each group, *n* = 3. Data are presented as mean ± standard deviation. *p < 0.05, ** *p* < 0.01, ***p < 0.001.

To investigate the impact of SOX8 on osteogenic differentiation of ligament fibroblasts, we generated SOX8‐overexpression and knockdown lentiviruses, which were successfully transfected into ligament fibroblasts. Fluorescence imaging confirmed efficient transfection (Figure S2A–D). SOX8 knockdown during osteogenic induction significantly elevated ALP activity and Alizarin Red staining intensity, in contrast to SOX8 overexpression, which lowered both (Figure 2A,B). Moreover, analyses using Western blot and qPCR showed that knocking down SOX8 led to a significant rise in the expression of osteogenic marker genes such as RUNX2, ALP, COL1A1, and OCN, while overexpressing SOX8 caused the opposite outcome (Figure 2C–E). These findings suggest that SOX8 serves as a negative regulator of osteogenic differentiation in ligament fibroblasts in vitro.

### 3.3 SOX8 Inhibits Ectopic Bone Formation In Vivo

**FIGURE 3:**
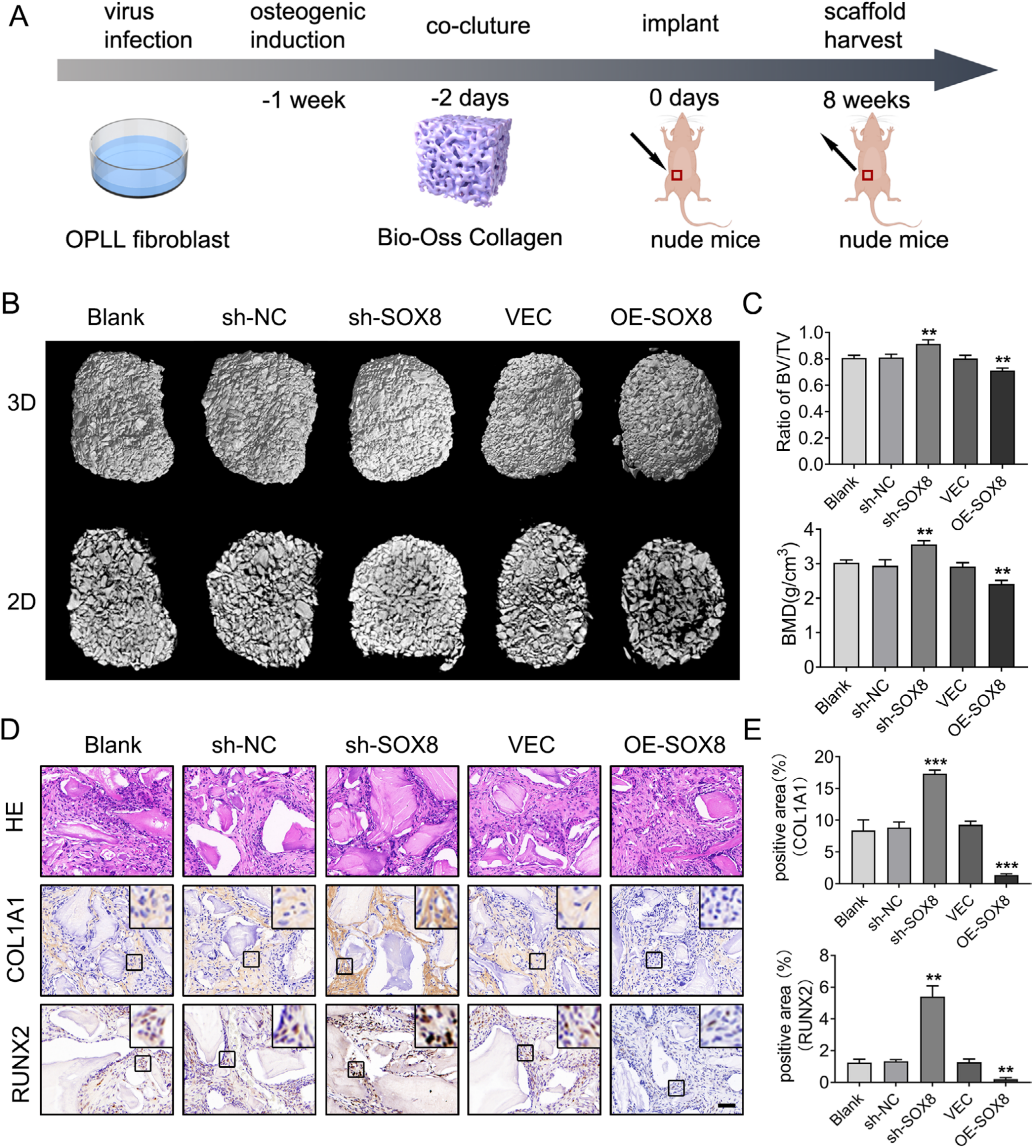
SOX8 inhibits bone formation in vivo. (A) Schematic representation of the ectopic bone formation assay in nude mice. (B) Representative 2D and 3D Micro‐CT images of Bio‐Oss Collagen scaffold implants after 8 weeks. (C) Quantitative analysis of bone volume/total volume (BV/TV) and bone mineral density (BMD) in the ectopic bone tissues, measured by Micro‐CT. (D) H&E staining and immunohistochemical staining for RUNX2 and COL1A1 in ectopic bone tissues. (E) Quantification of COL1A1 and RUNX2 expression based on immunohistochemical staining in ectopic bone tissues. For each group, *n* = 3. Data are presented as mean ± standard deviation. *p < 0.05, **p < 0.01, ***p < 0.001.

To explore the role of SOX8 in osteogenesis in vivo, we performed a subcutaneous ectopic bone formation assay using nude mice. Fibroblasts from OPLL patient ligaments were transfected with SOX8 knockdown (sh‐SOX8), SOX8 overexpression (OE‐SOX8), or control vectors (Blank, sh‐NC, and VEC) and underwent a week‐long osteogenic induction in vitro. After a two‐day co‐culture with Bio‐Oss collagen scaffolds, the cells were implanted under the skin in the right inguinal area of nude mice and allowed to grow for 8 weeks (Figure 3A). Micro‐CT analysis demonstrated that the sh‐SOX8 group had a significantly higher bone mass and volume of ectopic bone tissue than the other groups, as evidenced by both 2D and 3D reconstructed images. In contrast, the OE‐SOX8 group exhibited the lowest bone mass (Figure 3B). The sh‐SOX8 group exhibited a significant enhancement in bone volume/total volume (BV/TV) and bone mineral density (BMD) according to quantitative analysis, while the OE‐SOX8 group showed a significant reduction (Figure 3C). Histological examination via H&E staining showed increased new bone formation in the sh‐SOX8 group and reduced new bone formation in the OE‐SOX8 group (Figure 3D). Similarly, immunohistochemical staining indicated that SOX8 knockdown led to a higher number of COL1A1‐ and RUNX2‐positive cells, while SOX8 overexpression resulted in fewer COL1A1‐ and RUNX2‐positive cells (Figure 3D,E). In conclusion, these findings suggest that SOX8 inhibits ectopic bone formation in vivo.

### 3.4 TRIM25 Interacts With SOX8 and Is Highly Expressed in OPLL Patients

The previous findings identified SOX8 as a major negative regulator in the ectopic OPLL. However, the factors responsible for the reduced SOX8 expression in ossified ligaments remain unclear. Ubiquitination, a common post‐translational modification, regulates protein stability and expression levels through the ubiquitin‐proteasome system [34, 35, 36]. Our earlier results (Figure 1C–F) indicated that changes in SOX8 expression occur at the protein level, not the mRNA level, suggesting post‐translational regulation. Based on this, we proposed that ubiquitination could regulate SOX8 during the ectopic ossification process in OPLL.

**FIGURE 4:**
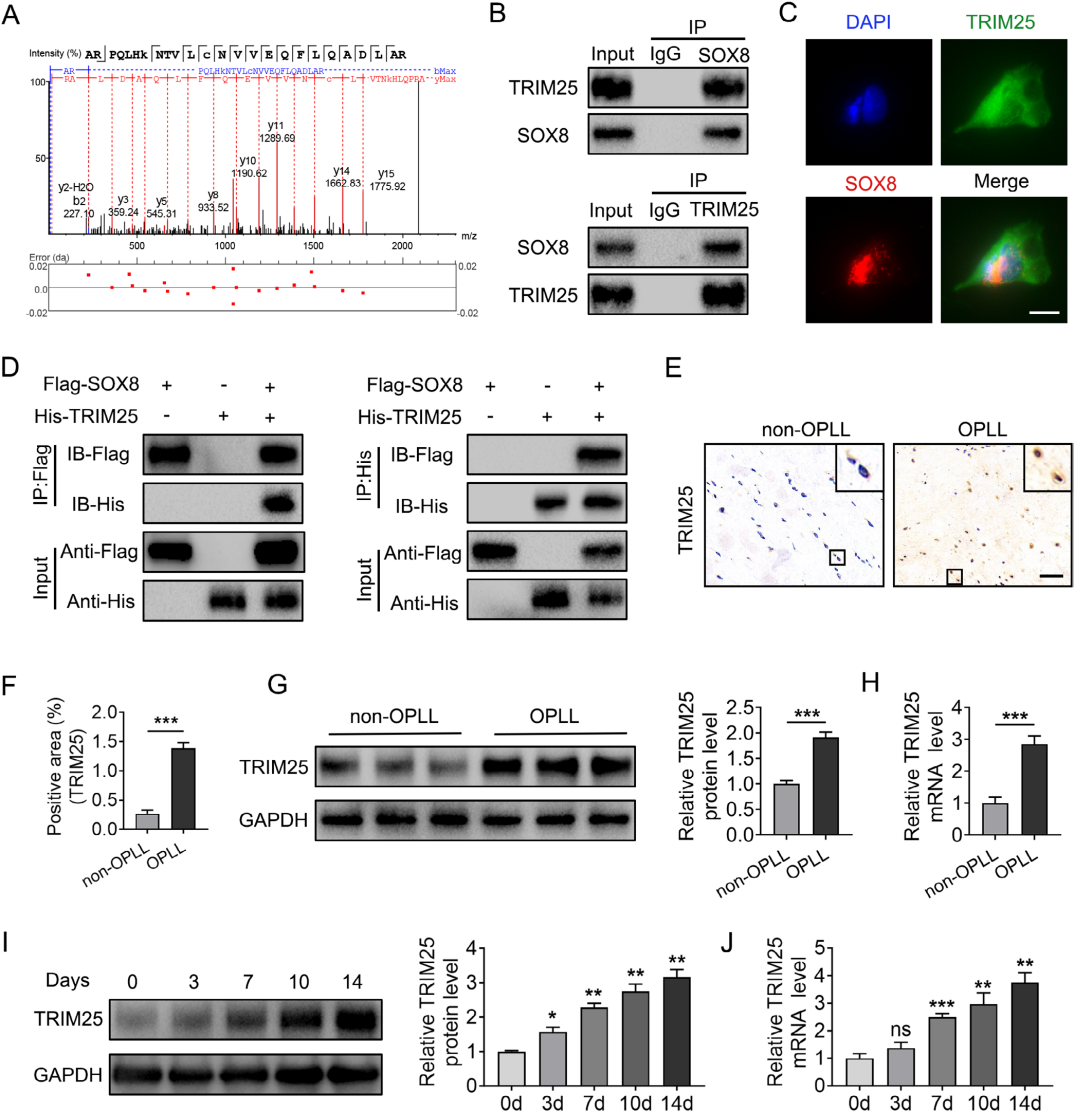
TRIM25 is significantly upregulated in OPLL patients and interacts with SOX8. (A) Proteomics analysis identifies the interaction between SOX8 and TRIM25. (B) Endogenous interaction of TRIM25 and SOX8 validated by co‐immunoprecipitation (CO‐IP) and Western blot in ligament fibroblasts. (C) Immunofluorescence showing colocalization of SOX8 (red) and TRIM25 (green) in fibroblasts. Nuclei were stained with DAPI (blue). Scale bar = 100 μm. (D) Exogenous interaction of TRIM25 and SOX8 confirmed by CO‐IP in HEK 293T cells. (E, F) Immunohistochemical staining for TRIM25 expression in ligament tissues from non‐OPLL and OPLL patients (*n* = 6). Scale bar = 100 μm. (G, H) TRIM25 expression differences in fibroblasts from non‐OPLL and OPLL patients analyzed by Western blot and qPCR. (I, J) TRIM25 expression during osteogenic differentiation of ligament fibroblasts assessed by Western blot and qPCR. Data are presented as mean ± standard deviation. *p < 0.05, **p < 0.01, ***p < 0.001 (all experiments performed in triplicate, otherwise specified).

Proteomics analysis revealed that TRIM25 is a potential E3 ubiquitin ligase interacting with SOX8 (Figure 4A). Co‐immunoprecipitation (CO‐IP) experiments confirmed this interaction. In ligament fibroblasts, TRIM25 was detected in complexes precipitated with SOX8 antibodies, and vice versa (Figure 4B). Additionally, in HEK 293T cells, exogenous CO‐IP assays confirmed the effective co‐precipitation of Flag‐tagged SOX8 with His‐tagged TRIM25 (Figure 4D). Immunofluorescence staining revealed colocalization of SOX8 and TRIM25 in cells (Figure 4C), further supporting the interaction between the two proteins.

We then examined TRIM25 expression in posterior longitudinal ligament tissues and fibroblasts. Immunohistochemistry indicated that ligament tissues from OPLL patients had a significantly higher expression of TRIM25 compared to those from non‐OPLL patients (*p* < 0.05; Figure 4E,F). Western blotting and qPCR analyses further confirmed elevated TRIM25 levels in fibroblasts from OPLL patients (Figure 4G,H). During osteogenic differentiation of ligament fibroblasts, TRIM25 expression increased over time, showing an inverse correlation with SOX8 levels (Figure 4I,J). These findings indicate that TRIM25 is highly expressed in posterior longitudinal ligament tissues and fibroblasts of OPLL patients and may regulate SOX8 protein levels, playing a critical role in OPLL‐related ectopic ossification.

### 3.5 TRIM25 Promotes Ubiquitination and Degradation of SOX8

**FIGURE 5:**
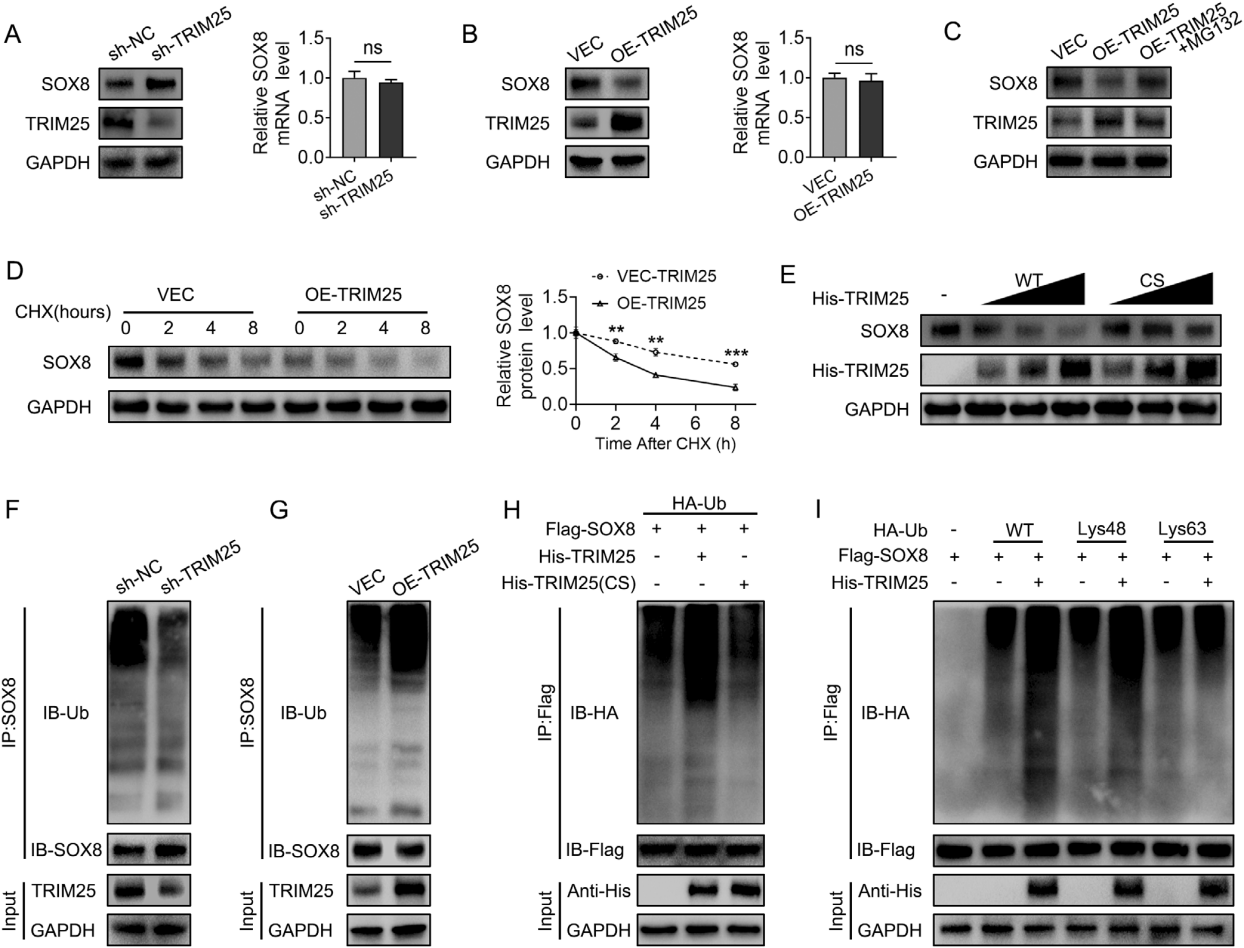
TRIM25 Promotes Ubiquitination and Accelerates Degradation of SOX8. (A) Western blot and qPCR analysis of TRIM25 and SOX8 expression in fibroblasts with TRIM25 knockdown. (B) Western blot and qPCR analysis of TRIM25 and SOX8 expression in fibroblasts overexpressing TRIM25. (C) Western blot analysis of SOX8 protein levels in TRIM25‐overexpressing fibroblasts treated with or without MG132. (D) Time‐course analysis of SOX8 protein degradation in TRIM25‐overexpressing fibroblasts treated with CHX. (E) Western blot of SOX8 and His‐TRIM25 expression in HEK 293T cells transfected with WT or CS TRIM25. (F) Endogenous SOX8 ubiquitination assessment in TRIM25‐knockdown fibroblasts. (G) Endogenous SOX8 ubiquitination assessment in TRIM25‐overexpressing fibroblasts. (H) Exogenous SOX8 ubiquitination analysis in HEK 293T cells co‐transfected with Flag‐SOX8, His‐TRIM25 (WT or CS), and HA‐Ub. (I) Analysis of K48 or K63‐linked ubiquitination of SOX8 in HEK 293T cells co‐transfected with Flag‐SOX8, His‐TRIM25, and lysine‐specific HA‐Ub. GAPDH was employed as an internal control. Data are given as mean ± standard deviation. *p < 0.05, **p < 0.01, ***p < 0.001 (all experiments were repeated three times).

To determine the influence of TRIM25 on SOX8 expression, we looked at how it alters SOX8 levels in fibroblasts from the posterior longitudinal ligament. TRIM25 knockdown led to an increase in SOX8 protein levels, while its overexpression resulted in a decrease, without altering mRNA expression (Figure 5A,B). The decrease in SOX8 protein levels triggered by TRIM25 overexpression was reversed with MG132 treatment, a proteasome inhibitor, indicating that TRIM25 regulates SOX8 at the protein level (Figure 5C). We then evaluated how TRIM25 affects the stability of the SOX8 protein. When cycloheximide (CHX), a protein synthesis inhibitor, was present, overexpressing TRIM25 notably sped up the degradation of SOX8 protein (Figure 5D). TRIM25 mutants (C50S/C53S, denoted as CS) are known to lose their E3 ubiquitin ligase activity without altering binding capacity [37]. When wild‐type TRIM25 (WT TRIM25) or its CS mutant (CS TRIM25) was transfected into HEK 293T cells, WT TRIM25 reduced SOX8 levels in a dose‐dependent manner, whereas CS TRIM25 had no significant effect (Figure 5E). This finding suggests that TRIM25 regulates SOX8 degradation via its E3 ubiquitin ligase activity.

We then investigated the effect of TRIM25 on SOX8 ubiquitination. TRIM25 knockdown significantly reduced SOX8 ubiquitination in ligament fibroblasts, whereas TRIM25 overexpression enhanced it (Figure 5F,G). Co‐transfection of HEK 293T cells with Flag‐SOX8, His‐TRIM25 (WT or CS), and HA‐Ub confirmed that WT TRIM25 promotes SOX8 ubiquitination, while CS TRIM25 does not (Figure 5H).

To further characterize the type of ubiquitin linkage involved, we examined whether TRIM25‐mediated ubiquitination of SOX8 involves K48 or K63‐linked polyubiquitination. Our results indicate that TRIM25 promotes K48‐linked polyubiquitination of SOX8, but not K63‐linked polyubiquitination (Figure 5I). This suggests that K48‐linked polyubiquitination is essential for TRIM25‐induced SOX8 degradation via the proteasomal pathway. In conclusion, TRIM25 promotes SOX8 ubiquitination and proteasomal degradation, thereby regulating SOX8 protein levels.

### 3.6 TRIM25 Promotes Osteogenic Differentiation of Posterior Longitudinal Ligament Fibroblasts by Downregulating SOX8

**FIGURE 6:**
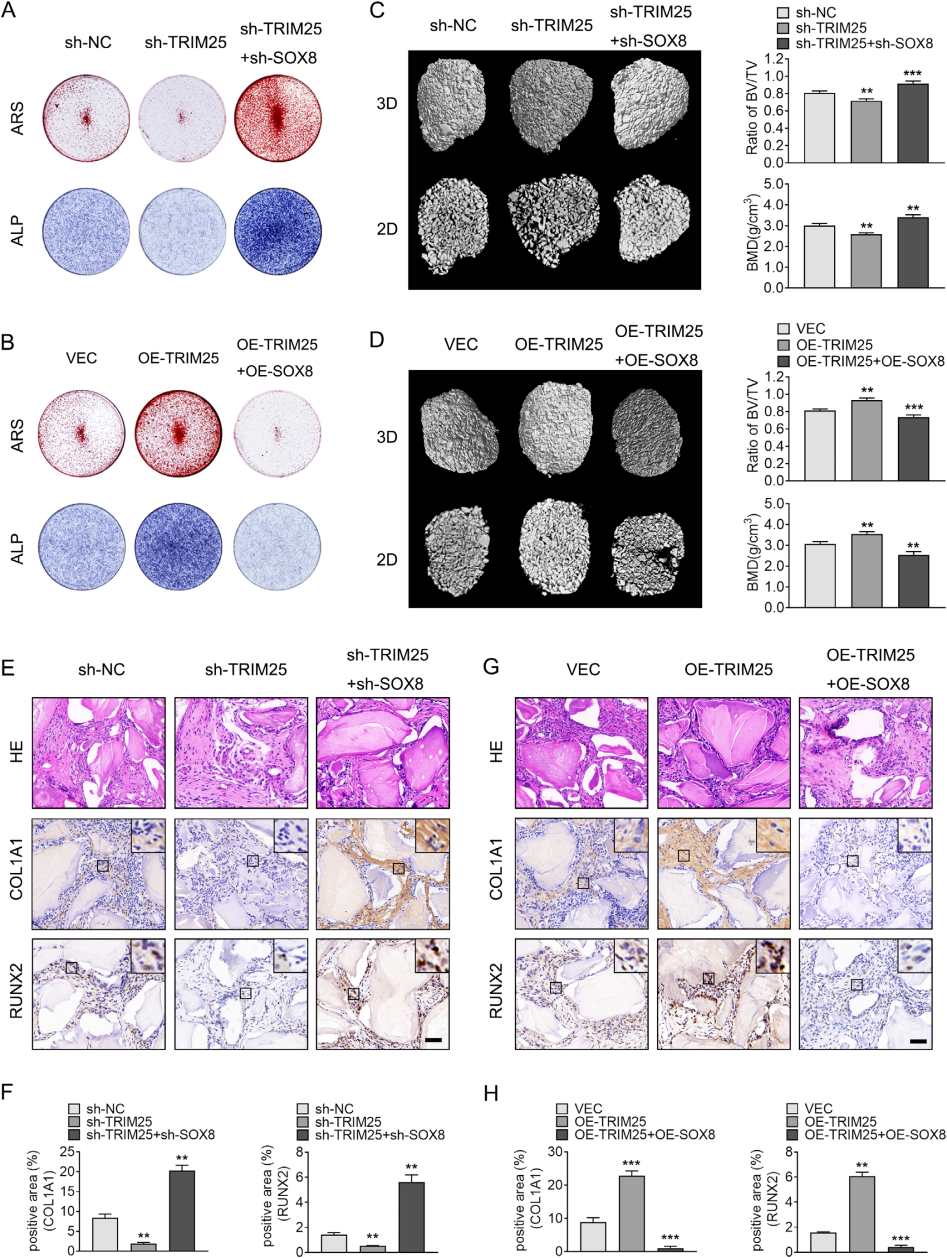
TRIM25 Promotes Osteogenic Differentiation of Ligament Fibroblasts by Regulating SOX8 Expression. (A) ALP activity and Alizarin Red staining in fibroblasts with TRIM25 knockdown, TRIM25 and SOX8 simultaneous knockdown, and control groups. (B) ALP activity and Alizarin Red staining in fibroblasts with TRIM25 overexpression, TRIM25 and SOX8 simultaneous overexpression, and control groups. (C) Micro‐CT 2D and 3D reconstructions, as well as analysis of BV/TV and BMD in Bio‐Oss collagen scaffolds implanted with fibroblasts from TRIM25 knockdown, TRIM25 and SOX8 simultaneous knockdown, and control groups. (D) Micro‐CT 2D and 3D reconstructions, along with BV/TV and BMD analysis, in Bio‐Oss collagen scaffolds implanted with fibroblasts from TRIM25 overexpression, TRIM25 and SOX8 simultaneous overexpression, and control groups. (E) H&E and immunohistochemical staining for RUNX2 and COL1A1 in fibroblasts with TRIM25 knockdown, TRIM25 and SOX8 simultaneous knockdown, and control groups. Quantification of RUNX2 and COL1A1 expression is shown in (F). (G) H&E and immunohistochemical staining for RUNX2 and COL1A1 in fibroblasts with TRIM25 overexpression, TRIM25 and SOX8 simultaneous overexpression, and control groups. Quantitative analysis of RUNX2 and COL1A1 expression is shown in (H). Scale bar = 100 μm. For each group, *n* = 3. Data are presented as mean ± standard deviation. *p < 0.05, **p < 0.01, ***p < 0.001.

We undertook functional rescue experiments both in vitro and in vivo to further examine the impact of SOX8 and TRIM25 on the osteogenic differentiation of ligament fibroblasts. In vitro, TRIM25 knockdown significantly reduced ALP activity and Alizarin Red staining intensity in fibroblasts. However, simultaneous knockdown of SOX8 reversed these effects (Figure 6A). Conversely, overexpression of SOX8 diminished the increase in ALP activity and Alizarin Red staining induced by TRIM25 overexpression (Figure 6B).

In vivo subcutaneous ectopic bone formation assays in nude mice showed that TRIM25 knockdown reduced bone formation, BV/TV, and BMD. However, simultaneous knockdown of SOX8 rescued these effects (Figure 6C). Likewise, overexpression of SOX8 reversed the enhanced bone formation, BV/TV, and BMD caused by TRIM25 overexpression (Figure 6D). The findings from H&E staining demonstrated that SOX8 knockdown restored the reduced new bone formation observed with TRIM25 knockdown, while SOX8 overexpression counteracted the increased bone formation induced by TRIM25 overexpression (Figure 6E,G). Consistent immunohistochemical staining for COL1A1 and RUNX2 showed similar trends. Knockdown of SOX8 reversed the decreased staining for RUNX2 and COL1A1 caused by TRIM25 knockdown, whereas overexpression of SOX8 reversed the increased staining induced by TRIM25 overexpression (Figure 6E–G). These findings indicate that TRIM25 promotes osteogenic differentiation of ligament fibroblasts by reducing SOX8 levels, both in vitro and in vivo.

### 3.7 SOX8 Regulates Osteogenic Differentiation of Posterior Longitudinal Ligament Fibroblasts by Promoting OSR2 Gene Transcription

**FIGURE 7:**
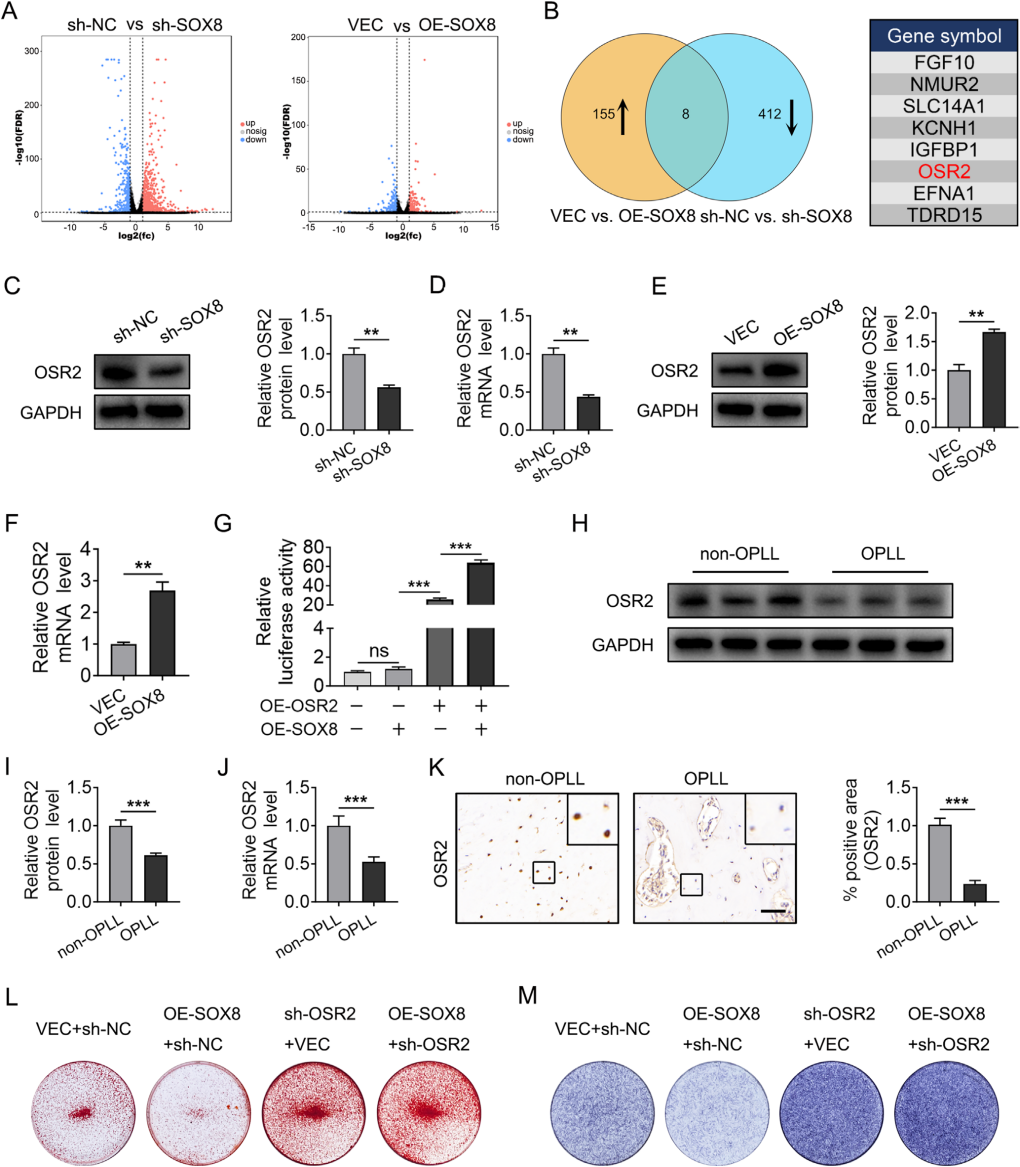
SOX8 Promotes Transcription of OSR2, Regulating Osteogenic Differentiation of Ligament Fibroblasts. (A) Volcano plot of differentially expressed genes following osteogenic differentiation in SOX8‐knockdown and SOX8‐overexpressing ligament fibroblasts compared to their respective controls (RNA‐seq). (B) Venn diagram illustrating the overlap of genes upregulated in SOX8 overexpression fibroblasts and downregulated in SOX8 knockdown fibroblasts. (C–F) Western blot and qPCR analysis of OSR2 expression in fibroblasts with SOX8 knockdown (C, D) or overexpression (E, F). (G) Dual‐luciferase reporter assay showing activation of the OSR2 promoter by SOX8 in HEK293T cells. (H–J) Western blot and qPCR analysis of OSR2 expression in fibroblasts from OPLL and non‐OPLL patients (*n* = 6). (K) Immunohistochemical staining of OSR2 expression in posterior longitudinal ligament tissues from OPLL and non‐OPLL patients, with relative quantification. *n* = 6, Scale bar = 100 μm. (L) Alizarin Red staining intensity in fibroblasts with SOX8 overexpression and OSR2 knockdown. (M) ALP activity in fibroblasts with SOX8 overexpression and OSR2 knockdown. All data are presented as mean ± standard deviation. *p < 0.05, **p < 0.01, ***p < 0.001 (all experiments performed in triplicate, otherwise specified).

SOX8, which is a transcription factor with an HMG domain, controls gene expression by attaching to the promoters of target genes [38, 39]. To explore how SOX8 influences the osteogenic differentiation of ligament fibroblasts, RNA sequencing was performed on fibroblasts with SOX8 overexpression or knockdown, alongside control groups. According to the RNA‐seq analysis, knocking down SOX8 led to the upregulation of 853 genes and the downregulation of 420 genes, whereas overexpressing SOX8 resulted in 163 genes being upregulated and 202 genes being downregulated (Figures 7A and S3A,B). A Venn diagram analysis intersecting the upregulated genes in SOX8‐overexpressing cells with the downregulated genes in SOX8 knockdown cells identified eight potential target genes (Figure 7B). Using the GTRD database, 16,348 possible SOX8 target genes were predicted (Additional file 1). Cross‐referencing these data identified three overlapping genes: OSR2, EFNA1, and TDRD15. Among these, OSR2—a zinc‐finger transcription factor known to promote cartilage development and inhibit mesenchymal stem cell osteogenesis [40, 41, 42, 43]—was prioritized for further investigation.

Western blot and qPCR analyses showed that SOX8 knockdown reduced both OSR2 protein (Figure 7C) and mRNA levels (Figure 7D), whereas SOX8 overexpression increased OSR2 protein (Figure 7E) and mRNA levels (Figure 7F), consistent with the RNA‐seq results. To confirm that SOX8 directly regulates OSR2 transcription, a dual‐luciferase reporter assay was performed. SOX8 overexpression significantly enhanced OSR2 promoter activity in HEK293T cells, validating OSR2 as a direct transcriptional target of SOX8 (Figure 7G). Immunohistochemical staining further revealed that OSR2 expression was significantly lower in posterior longitudinal ligament tissues from OPLL patients compared to non‐OPLL patients (*p* < 0.05, Figure 7K). This finding was corroborated by Western blot and qPCR analyses of fibroblasts derived from OPLL and non‐OPLL tissues (Figure 7H–J).

To investigate the functional relationship between SOX8 and OSR2, rescue experiments were performed. Overexpression of SOX8 significantly reduced Alizarin Red staining intensity, but this effect was reversed by OSR2 knockdown (Figure 7L). Similarly, SOX8 overexpression reduced alkaline phosphatase (ALP) activity, an effect that was restored by OSR2 knockdown (Figure 7M). Together, the data indicate that SOX8 promotes OSR2 transcription, which regulates the osteogenic differentiation of ligament fibroblasts.

## 4 Discussion

In this investigation, it was found that SOX8 expression was considerably decreased in PLL tissues and fibroblasts from OPLL patients, suggesting a key role for SOX8 in the ectopic ossification process in OPLL. Through a series of in vitro and in vivo gain‐ and loss‐of‐function experiments, we confirmed that SOX8 inhibits osteogenic differentiation of ligament fibroblasts and suppresses ectopic ossification in nude mice. Additionally, proteomics analysis and functional rescue experiments revealed that the E3 ubiquitin ligase TRIM25 binds to SOX8, promoting its degradation via the ubiquitin‐proteasome system, thereby reducing its expression and affecting the osteogenic differentiation of ligament fibroblasts. Combining transcriptomic sequencing, GTRD database analysis, and dual‐luciferase reporter assays, we identified OSR2 as a transcriptional target gene of SOX8. SOX8 enhances OSR2 gene transcription by upregulating the activity of the OSR2 promoter, thereby regulating the ossification process of ligament fibroblasts.

OPLL is a multifactorial disease characterized by ectopic ossification of the spinal PLL. Gaining insight into the mechanisms and factors affecting the ossification of the posterior longitudinal ligament can aid in creating non‐surgical treatments for OPLL. There has been a growing emphasis in recent studies on the contribution of PLL‐derived fibroblasts to the ectopic ossification in OPLL. These fibroblasts possess stem‐like characteristics and differentiation potential, and the regulatory mechanisms of their osteogenic differentiation are key to understanding the potential pathogenesis of OPLL [9, 10, 11]. Compared to non‐OPLL patients, ligament fibroblasts from OPLL patients exhibit higher ossification potential and tend to differentiate into osteogenic lineages. The propensity for osteogenesis could be a critical component in the development of abnormal bone formation in OPLL. Our immunohistochemical results also showed increased expression of osteogenic markers, such as COL1A1 and RUNX2, in PLL tissues from OPLL patients (Figure 1B), consistent with their higher ossification potential. At the same time, we observed a downregulation of SOX8 in ossified PLL tissues and in cultured fibroblasts, with SOX8 expression progressively decreasing during the osteogenic differentiation of fibroblasts (Figure 1B–F). These findings imply that SOX8 may be an important factor in the ossification process in OPLL.

Previous studies have mainly highlighted the roles of SOX8 in various physiological and pathological processes, such as chemotherapy resistance, tumor development, reproductive system development, and nervous system development [44, 45, 46, 47]. Some research has also explored the role of SOX8 in cartilage differentiation [20, 48]. However, its role in ectopic ossification has not been fully clarified. Our study indicates that modulation of SOX8 expression significantly affects the osteogenic capacity of ligament fibroblasts (Figure 2A–E). Overexpression of SOX8 significantly inhibited osteogenic differentiation of ligament fibroblasts and ectopic ossification in nude mice, while SOX8 knockdown had the opposite effect (Figure 3B–E). These findings emphasize the inhibitory effect of SOX8 on OPLL progression.

Interestingly, SOX8 expression was primarily lower at the protein level in OPLL‐derived fibroblasts compared to non‐OPLL‐derived ligament fibroblasts, rather than at the mRNA level. This suggests that the alteration of SOX8 expression may be regulated by post‐translational modifications. Ubiquitination is a common post‐translational modification that primarily regulates protein stability, activity, subcellular localization, and other functions, without affecting mRNA expression. Ubiquitination is essential for controlling the activity and stability of SOX proteins [49]. For instance, the regulation of thyroid cancer cell migration and invasion by TRIM30 involves targeting SOX17 for K48‐linked polyubiquitination [50], while E3 ligase E6‐AP affects SOX9 expression through ubiquitin‐mediated degradation, influencing cartilage development [51]. As members of the SOX E subgroup, SOX8, SOX9, and SOX10 share similar structures and genomic compositions. However, whether SOX8 itself is regulated by ubiquitination has not been previously established. In our study, we demonstrated that TRIM25 directly interacts with SOX8 and facilitates its ubiquitination‐mediated degradation through the ubiquitin‐proteasome pathway (Figure 5A–H). Our mechanistic studies further elucidated the specific ubiquitination pattern of SOX8 mediated by TRIM25. Among the two key ubiquitination linkage types, K48‐linked polyubiquitination is typically responsible for substrate proteins for degradation via the proteasome, whereas K63‐linked polyubiquitination is mainly involved in modulating protein trafficking, endocytosis, and activity regulation [52]. Notably, our findings demonstrate that TRIM25 specifically induces K48‐linked, but not K63‐linked, polyubiquitination of SOX8 (Figure 5I). These results establish TRIM25 as a negative regulator of SOX8 stability through K48‐linked ubiquitination and subsequent proteasomal degradation, revealing a novel post‐translational regulatory mechanism controlling SOX8 homeostasis.

The TRIM family proteins are crucial for several important cellular mechanisms, such as cell proliferation, differentiation, apoptosis, cancer, and innate immunity against viral infections [53]. Several studies have also reported that TRIM proteins regulate osteogenesis. For example, TRIM21 hinders the osteogenic differentiation of bone marrow mesenchymal stem cells by enhancing K48‐linked ubiquitination of Akt [54]. Other TRIM proteins, such as TRIM38, TRIM33, and TRIM16, have been reported to promote osteogenesis of osteoblasts or bone marrow mesenchymal stem cells [55, 56, 57]. TRIM25, which is part of the TRIM family, shows high expression levels in osteoblasts and might be involved in their differentiation [58]. In our study, overexpression of TRIM25 significantly promoted ALP activity and Alizarin Red staining intensity, while knockdown of TRIM25 resulted in the opposite effects. Additionally, ectopic ossification in nude mice further confirmed the osteogenic effect of TRIM25. Further functional rescue experiments confirmed that SOX8 overexpression reversed the osteogenic effect induced by TRIM25 overexpression, while SOX8 knockdown reversed the inhibitory effect of TRIM25 knockdown (Figure 6A–H). This suggests that TRIM25 influences the osteogenic differentiation of ligament fibroblasts by ubiquitinating SOX8, which in turn impacts the development of OPLL.

To further explore the downstream mechanisms of SOX8 in OPLL progression, we performed transcriptomic sequencing on fibroblasts with SOX8 overexpression and knockdown. Given that SOX family members are classic gene transcription regulators, we identified common differentially expressed genes and combined them with the GTRD database and dual‐luciferase reporter assays. Ultimately, we identified OSR2 as a transcriptional target of SOX8 (Figure 7A–G). OSR2, which promotes cartilage differentiation, helps maintain the homeostasis of chondrocytes [40]. In OSR2^RFP/−^ mutant embryos, expression of key signaling molecules and transcription factors involved in osteoblast differentiation, such as RUNXnx2, SP7, and BMP3, is upregulated [42]. Previous studies have suggested that OSR2 is a negative regulator of osteogenesis and bone formation [43]. In our study, functional rescue experiments confirmed that OSR2 knockdown not only restored the reduced Alizarin Red staining intensity but also restored the decrease in ALP activity following SOX8 overexpression (Figure 7L,M). More importantly, we found that OSR2 levels were reduced in PLL tissues and fibroblasts from OPLL patients (Figure 7H–K), consistent with the trend observed for SOX8 expression. This indicates that SOX8 mainly controls the ossification of ligament fibroblasts by influencing OSR2 expression.

However, there are some limitations to this study. First, while subcutaneous ossification models in nude mice successfully demonstrated the role of the TRIM25/SOX8/OSR2 axis in OPLL, it cannot fully recapitulate the complex mechanical and immunological microenvironment of human spinal ligaments, which are critical to OPLL pathogenesis. Future studies employing the tiptoe walking (ttw) mouse model—currently the most widely used and pathologically relevant OPLL animal system—would provide more physiologically representative evidence to validate these mechanisms. Second, OPLL is a multifactorial disease involving genetic, environmental, and mechanical interactions. The potential crosstalk between our identified pathway and other known OPLL regulators (e.g., NOTCH, BMP, Wnt, etc.) remains to be explored. Targeted studies dissecting these interactions may reveal novel therapeutic nodes. Additionally, the microRNA regulatory network and inflammatory responses are critically involved in the pathogenesis of OPLL. In addition to its canonical ubiquitination regulatory function, TRIM25 has been demonstrated to interact with multiple microRNAs (e.g., microRNA‐30a) and inflammatory pathways [59, 60, 61]. Future studies should further elucidate its upstream regulatory mechanisms.

**FIGURE 8:**
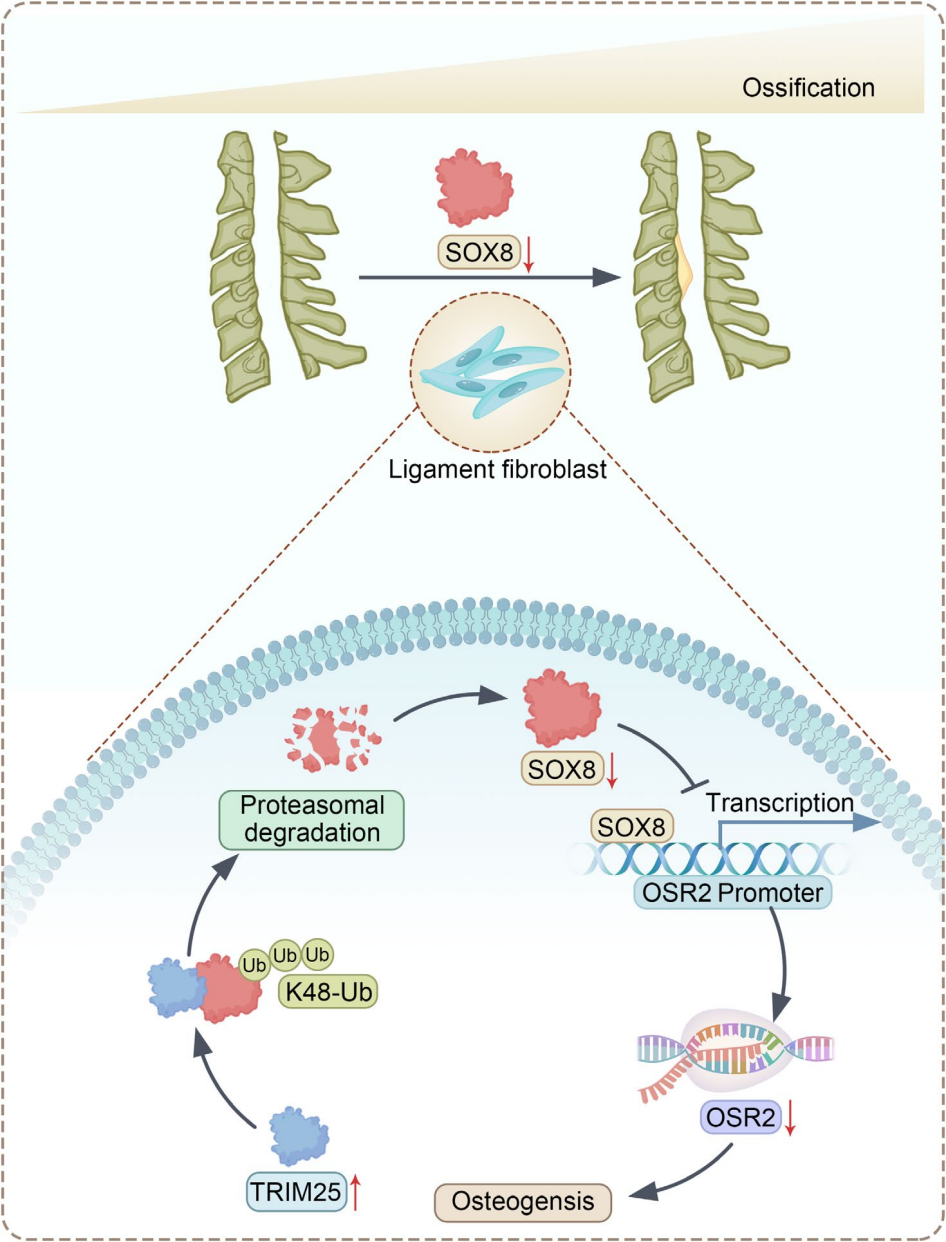
Schematic diagram of the regulation of Ectopic Ossification in OPLL by the TRIM25/SOX8/OSR2 axis.

In conclusion, this study highlights the critical role of the TRIM25/SOX8/OSR2 axis in the mechanism of ectopic ossification in OPLL (Figure 8), providing new insights into the pathogenesis of this disease. These findings offer a potential foundation for the development of targeted therapeutic strategies, which may hold significant clinical translational value.

## Author Contributions

**Z.W., Y.T., C.G., X.C.:** conceptualization. **X.C., S.Z.:** supervision. **Z.W., C.G., M.L., Z.H.W., Q.Z.:** data curation. **M.L., Z.H.W., Q.Z.:** formal analysis. **X.C., Y.T.:** funding acquisition. **Z.W., Y.T., C.G., M.L.:** investigation. **Z.W., M.L., Z.H.W., Q.Z.:** methodology. **Z.W., Y.T., C.G.:** validation. **Z.W.:** writing – original draft. **Y.T., C.G., S.Z., X.C.:** writing – review and editing.

## Ethics Statement

The study protocol for clinical sample collection was approved by the Committee on Ethics of Medicine, Navy Medical University, PLA.

## Consent

Informed consent was obtained from all patients prior to surgery. All animal experiments were conducted in compliance with ethical guidelines and received formal approval from the Committee on Ethics of Medicine, Navy Medical University, PLA.

## Conflicts of Interest

The authors declare no conflicts of interest.

## Supporting information


**Data S1:** Supporting Information.

**Table S1:** List of Primary Antibodies.
**Table S2:** The Sequences of Primers.
**Table S3:** Characteristics of Patients.
**Figure S1:** Extraction and Culture of Ligament Fibroblasts.(A) Fibroblast extraction using the tissue block attachment method. (B) Morphology of fibroblasts migrating from the edge of the tissue block. (C) Appearance of passaged fibroblasts. Scale bar = 100 μm.
**Figure S2:** Verification of SOX8 Lentiviral Transfection Efficiency.(A) Fluorescence microscopy image showing successful transfection of SOX8 lentivirus into ligament fibroblasts. (B) qPCR analysis confirming transfection efficiency for SOX8 knockdown and overexpression lentiviruses. (C) Western blot analysis validating transfection efficiency for SOX8 knockdown lentivirus. (D) Western blot analysis validating transfection efficiency for SOX8 overexpression lentivirus. Scale bar = 100 μm. All data are presented as mean ± standard deviation. *p < 0.05, **p < 0.01, ***p < 0.001 (experiments repeated three times).
**Figure S3:** Heatmap of Differentially Expressed Genes in SOX8‐Knockdown and SOX8‐Overexpressing Fibroblasts.

## Data Availability

The data that support the findings of this study are available from the corresponding author upon reasonable request.
