# Supplementary material for: TRIM25‐Mediated Ubiquitination and Degradation of SOX8 Promotes Ligament Fibroblast Osteogenic Differentiation and Regulates OPLL Progression by Inhibiting OSR2 Transcription
Source: JOR Spine. 2025 Sep 5;8(3):e70112. doi: 10.1002/jsp2.70112 (PMC12412890; doi:10.1002/jsp2.70112)
Supplement: Supplementary file 2 — Table S1: List of Primary Antibodies. Table S2: The Sequences of Primers. Table S3: Characteristics of Patients. Figure S1: Extraction and Culture of Ligament Fibroblasts.(A) Fibroblast extraction using the tissue block attachment method. (B) Morphology of fibroblasts migrating from the edge of the tissue block. (C) Appearance of passaged fibroblasts. Scale bar = 100 μm. Figure S2: Verification of SOX8 Lentiviral Transfection Efficiency.(A) Fluorescence microscopy image showing successful transfection of SOX8 lentivirus into ligament fibroblasts. (B) qPCR analysis confirming transfection efficiency for SOX8 knockdown and overexpression lentiviruses. (C) Western blot analysis validating transfection efficiency for SOX8 knockdown lentivirus. (D) Western blot analysis validating transfection efficiency for SOX8 overexpression lentivirus. Scale bar = 100 μm. All data are presented as mean ± standard deviation. *p < 0.05, **p < 0.01, ***p < 0.001 (experiments repeated three times). Figure S3: Heatmap of Differentially Expressed Genes in SOX8‐Knockdown and SOX8‐Overexpressing Fibroblasts. [file JSP2-8-e70112-s001.docx]

**Supplementary Table S1. List of Primary Antibodies**

| **Primary antibodies** | **Concentration** | **Company** | | **Catalog Number** |
| --- | --- | --- | --- | --- |
| SOX8  SOX8  TRIM25  TRIM25  RUNX2  RUNX2  ALP  COL1A1  COL1A1  OCN  Flag | WB (1:1000)  IHC (1:50)  WB (1:2000)  IHC (1:100)  WB (1:1000)  IHC (1:100)  WB (1:1000)  WB (1:1000)  IHC (1:100)  WB (1:1000)  WB (1:1000) | | Santa cruz  Proteintech  Abcam  Abcam  ABclonal  ABclonal  ABclonal  Beyotime  CST  ABclonal  ABclonal | sc-374446  20627-1-AP  Ab167154  Ab167154  A11753  A2851  A0514  AF1840  72026  A20800  AE005  66005-1-IG  51064-2-AP  10201-2-AP  A18178  DF4670  A19056 |
| His  HA  Ubiquitin  OSR2  OSR2  GAPDH | WB (1:1000)  WB (1:2000)  WB (1:1000)  WB (1:1000)  IHC (1:100)  WB（1:50000） | | Proteintech  Proteintech  Proteintech  ABclonal  Affinity Biosciences  ABclonal |  |

**Supplementary Table S2. The Sequences of Primers**

| **Primer Name** | **Sequence (5'to 3')** |
| --- | --- |
| SOX8  RUNX2  ALP  OCN  COL1A1  TRIM25  OSR2  GAPDH | F: GAGCTTGGCAACCGAAAACCC  R: GAGTCCTGCTTCCCAAACCC  F: TACTATGGCACTTCGTCAGGA  R: TTTAATAGCGTGCTGCCATTCGAG  F: AGGCTTGCCCCAAATCTCAA  R: GACCTTTGGCTCTCGACCAG  F: GGCAGCGAGGTAGTGAAGAGA  R: CTCCTGAAAGCCGATGTGG  F: GGCCAAGACGAAGACATCCCA  R: TCACGTCATCGCACAACACC  F: CGTGCTTCTCAACTGTGACCA  R: TACAAAGCCTCAGTAAAGTCCAC  F: TCCGCCTAAGATGGGAGACC  R: GGTAAAGTGTCTGCCGCAAAA  F: GCACCGTCAAGGCTGAGAAC  R: TGGTGAAGACGCCAGTGGA |

**Supplementary Table S3. Characteristics of Patients**

| **Group** | **Sex** | **Age** | **Diagnosis** | **Surgical level** |
| --- | --- | --- | --- | --- |
| OPLL1 | Male | 63 | OPLL | C6-7 |
| OPLL2  OPLL3  OPLL4  OPLL5  OPLL6  OPLL7  OPLL8  OPLL9  OPLL10  OPLL11  OPLL12  non-OPLL1  non-OPLL2  non-OPLL3  non-OPLL4  non-OPLL5  non-OPLL6  non-OPLL7  non-OPLL8  non-OPLL9  non-OPLL10  non-OPLL11  non-OPLL12  P value | Female  Female  Male  Female  Male  Male  Male  Male  Male  Female  Female  Female  Male  Male  Female  Male  Male  Female  Female  Male  Female  Male  Female  0.682 | 57  52  67  60  49  51  75  49  71  70  60  70  65  52  48  57  64  68  56  50  68  52  61  0.589 | OPLL  OPLL  OPLL  OPLL  OPLL  OPLL  OPLL  OPLL  OPLL  OPLL  OPLL  cervical spine trauma  cervical spine trauma  cervical spine trauma  cervical spine trauma  cervical spine trauma  cervical spine trauma  cervical spine trauma  cervical spine trauma  cervical spine trauma  cervical spine trauma  cervical spine trauma  cervical spine trauma  - | C5  C5  C6-7  C4-5  C5  C5-6  C5  C6  C5  C6-7  C6  C4  C6  C5  C4  C4  C6  C5  C6  C5  C4  C5  C5  - |

OPLL: ossification of the posterior longitudinal ligament

**Supplementary Figures**


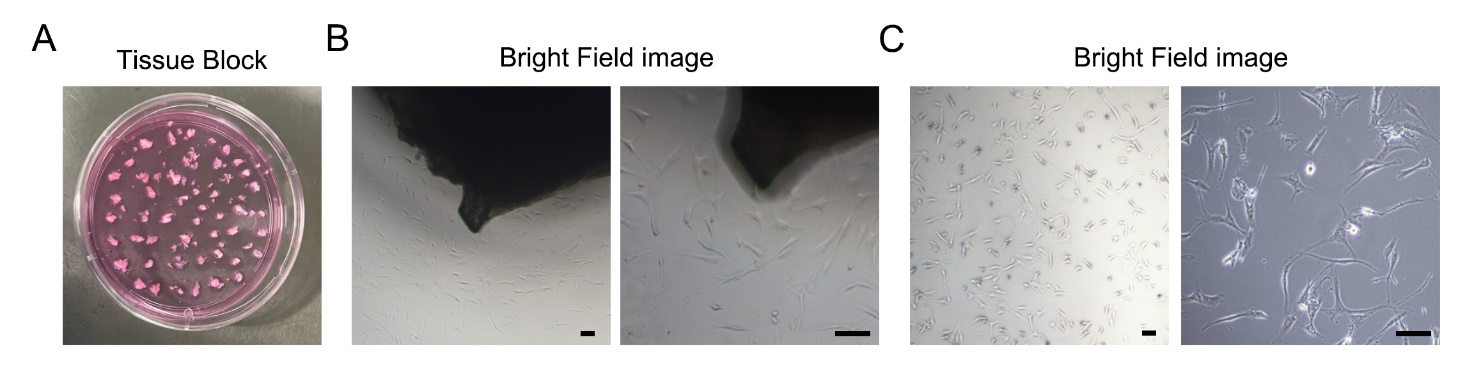


**Figure S1 Extraction and Culture of Ligament Fibroblasts.**
**(A)** Fibroblast extraction using the tissue block attachment method. **(B)** Morphology of fibroblasts migrating from the edge of the tissue block. **(C)** Appearance of passaged fibroblasts. Scale bar = 100 µm.


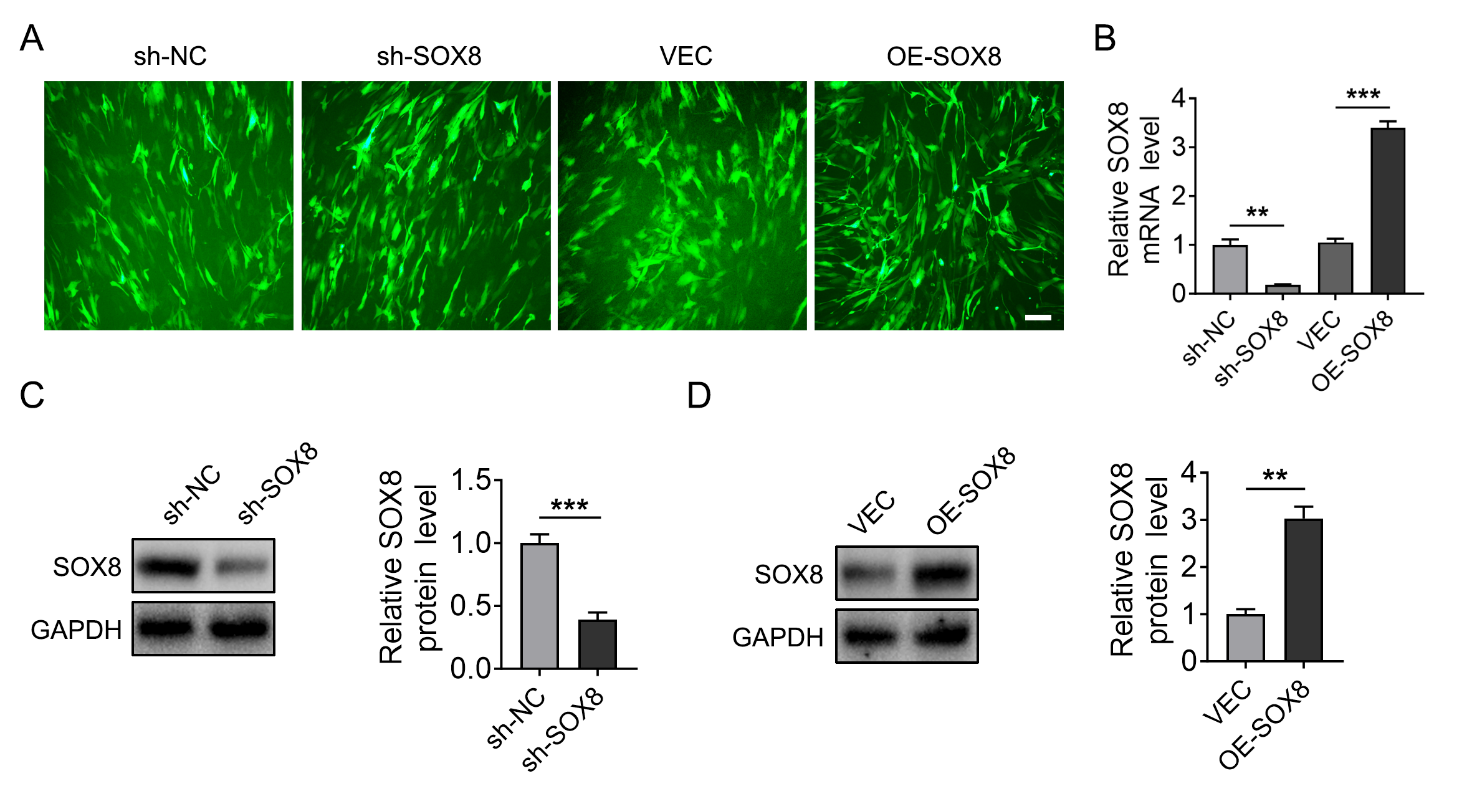


**Figure S2 Verification of SOX8 Lentiviral Transfection Efficiency.**
**(A)** Fluorescence microscopy image showing successful transfection of SOX8 lentivirus into ligament fibroblasts. **(B)** qPCR analysis confirming transfection efficiency for SOX8 knockdown and overexpression lentiviruses. **(C)** Western blot analysis validating transfection efficiency for SOX8 knockdown lentivirus. **(D)** Western blot analysis validating transfection efficiency for SOX8 overexpression lentivirus. Scale bar = 100 µm. All data are presented as mean ± standard deviation. **P* < 0.05, ***P* < 0.01, ****P* < 0.001 (experiments repeated three times).


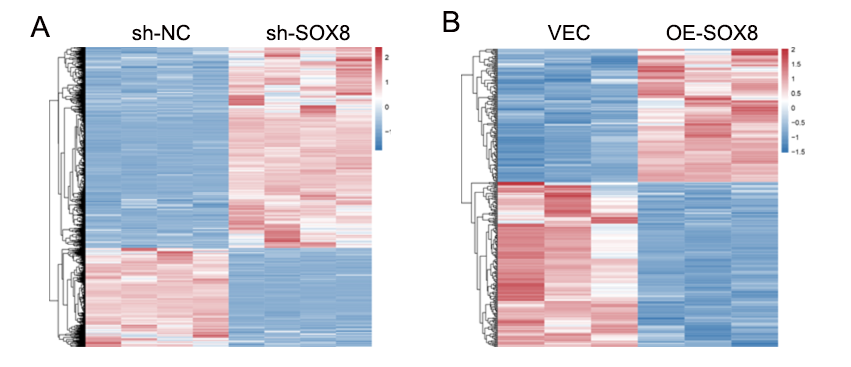


**Figure S3** **Heatmap of Differentially Expressed Genes in SOX8-Knockdown and SOX8-Overexpressing Fibroblasts.**

**(A)** Heatmap showing differentially expressed genes identified by RNA-seq in SOX8-knockdown fibroblasts compared to control cells. **(B)** Heatmap showing differentially expressed genes identified by RNA-seq in SOX8-overexpressing fibroblasts compared to control cells.
